# Downregulated PLAU alleviates acute rejection after liver transplantation by targeting Ptgs2 in macrophages

**DOI:** 10.3389/fimmu.2026.1779520

**Published:** 2026-03-31

**Authors:** Qiong Qin, Zhe-Chao Wang, Shi-Ming Jiang, Jun-Hua Gong, Yong Zhou, Zhao-Wei Wu, Yong Chen

**Affiliations:** 1 Department of Hepatobiliary Surgery, The First Affiliated Hospital of Chongqing Medical University, Chongqing, China; 2 Department of Hepatobiliary Surgery, Chongqing University Jiangjin Hospital, Chongqing, China; 3 Department of Hepatobiliary Surgery, The Second Affiliated Hospital of Chongqing Medical University, Chongqing, China; 4 Department of Hepatobiliary Surgery, Chongqing University FuLing Hospital, Chongqing, China

**Keywords:** acute rejection, Akt/NF-κB pathway, liver transplantation, PLAU, PTGS2

## Abstract

**Background:**

Acute rejection (AR) is a major determinant of poor prognosis after liver transplantation (LT), and macrophage M1-polarization can induce AR. Urokinase-type plasminogen activator (PLAU) has been implicated in the regulation of several liver diseases, but its role in AR remains unclear.

**Methods:**

In this study, through bioinformatics analysis and transcriptome sequencing, we find that PLAU and prostaglandin-endoperoxide synthase 2 (Ptgs2) can regulate macrophage polarization. To elucidate the effects of PLAU and Ptgs2 on AR, dynamic changes in PLAU and Ptgs2 expression are detected in both peripheral blood mononuclear cells (PBMCs) from clinical LT patients and hepatic macrophages from LT rats. Subsequently, to investigate the specific regulatory roles in macrophage M1-polarization and AR, downregulating PLAU and Ptgs2 rescue experiments are conducted *in vivo* and *in vitro*. Finally, potential signaling pathways are further identified through KEGG enrichment analysis.

**Results:**

The expression of PLAU and Ptgs2 is increased in PBMCs from LT patients and in macrophages from LT rats, and most significantly on postoperative day 7. Downregulating PLAU alleviates AR and suppresses M1-polarization of macrophages. However, Ptgs2 rescue exacerbates AR and promotes M1-polarization of macrophages. The potential mechanism involves regulating the Protein kinase B/Nuclear factor kappa B (AKT/NF-κB) pathway.

**Conclusion:**

In summary, downregulated the PLAU/Ptgs2 axis can attenuate AR by regulating macrophage polarization, offering a promising new therapeutic target for preventing and treating AR.

## Introduction

Liver transplantation (LT) remains the only effective treatment strategy for end-stage liver disease (1, 2), such as hepatocellular carcinoma (HCC) (3). With ongoing refinements in surgical techniques, acute rejection (AR) has emerged as a major cause of poor post-transplant prognosis, occurring in up to 50% of recipients within five years, most commonly during the first week after surgery (4, 5). Immunosuppressants, such as tacrolimus (Tac), have become the first-line treatment for AR; however, long-term administration not only imposes a substantial economic burden but also increases adverse reactions such as organ toxicity, infections, malignancies, and graft dysfunction (6, 7). Therefore, successfully identifying new targets for AR treatment and elucidating the potential mechanisms will bring significant benefits to patients.

Macrophages, as secondary defense of the immune system, including kupffer cells (KCs) in the liver and monocytes in peripheral blood mononuclear cells (PBMCs), contribute to AR through pathogen phagocytosis, antigen presentation, and cytokine secretion, etc. (8). Among five types of activated macrophages, M1-polarized macrophages secrete pro-inflammatory cytokines such as IL-1β, IL-6, TNF-α, and iNOS, inducing immune rejection; M2-polarized macrophages secrete anti-inflammatory cytokines such as IL-10 and TGF-β, inducing immune tolerance (9). Our previous research has confirmed that inhibiting macrophage M1-polarization and promoting M2-polarization can effectively mitigate AR (10, 11). Additionally, inhibiting macrophage pyroptosis can significantly alleviate inflammatory responses (12) and reduce rejection reactions.

Urokinase-type plasminogen activator (PLAU) participates in physiological activities, including immune responses, by converting plasminogen into plasmin (13). PLAU is expressed at low levels in physiologically normal macrophages, while markedly increases under stimulation of cytokines, hormones, growth factors, etc. (14). Altered PLAU expression has been documented in several pathological conditions, including pulpitis (15), tumors (16), thrombotic disorders (17), aging (18), and Alzheimer’s disease (AD) (19), involving the Protein kinase B/Nuclear factor kappa B (AKT/NF-κB) pathway (14, 15). Moreover, PLAU has been confirmed to mitigate liver fibrosis and cirrhosis by facilitating extracellular matrix degradation (13). Early research has reported an association between elevated PLAU levels post-LT and increased bleeding risk (20), but its potential function in AR remains unclear.

Prostaglandin-endoperoxide synthase 2 (Ptgs2) is widely expressed in cells and catalyzes the conversion of arachidonic acid to prostaglandin H2, and the expression increases significantly during tissue inflammation and injury (21). Abnormal expressions of Ptgs2 is implicated in cancer (22), neurodegenerative disorders (23), and cardiovascular diseases (24), serving as key regulators in multiple pathologies. While Ptgs2 has been linked to ischemia-reperfusion injury (I/RI) in LT (25), its role in AR remains unexplored. Several studies have identified the AKT/NF-κB pathway participate in macrophage polarization in conditions such as abdominal aortic aneurysm (26), cancer (27), pneumonia (28), and pulpitis (15). However, research on AR in LT has primarily focused on NF-κB in macrophage polarization (29, 30), while investigations into the AKT/NF-κB remain limited.

In this study, we investigate the regulatory role and mechanisms of the PLAU/Ptgs2 axis in macrophage M1-polarization and AR, aiming to identify new targets for the prevention and treatment of AR.

## Materials and methods

### Ethics statement

This study was approved by the Clinical Research Ethics Review Committee of the First Affiliated Hospital of Chongqing Medical University (Approval number: 2024-487-01) and Institutional Animal Care and Use of Chongqing Medica University (Approval number: IACUC-CQMU-2024-0908). All human studies obtained informed consent from participants and complied with the Declaration of Helsinki.

### Bioinformatics analysis and transcriptome sequencing

In bioinformatics analysis, RNA sequencing data from 17 clinical liver tissue samples post-transplantation obtained from the Gene Expression Omnibus (GEO) database were analyzed, including 8 tolerance cases (Tol) and 9 rejection cases (NonT). Differentially expressed genes (DEGs) between Tol and NonT cases were identified using the edgeR package with the adjusted *P*-value < 0.05. Ultimately, only DEGs with |log2FC| > 0.58 were included as candidate genes of interest. Weighted gene co-expression network analysis (WGCNA) was performed with a soft threshold *β* = 9 to construct adjacency and topological overlap matrices, from which macrophage-associated genes among the DEGs were extracted. Then, using Pearson correlation analysis, genes were grouped into distinct modules based on their correlation strength with different cell types and visualized as a heatmap. Subsequently, core genes meeting both criteria were selected: (1) trait-relatedness absolute value > 0.4 (indicating strong association with M1 or M2 macrophages); (2) module membership > 0.8 (reflecting high module centrality). Using KEGG enrichment analysis, PLAU was selected for investigation, as its role in AR remained unclear but it participated in NF-κB pathway, which had been confirmed to be involved in the M1-polarization of macrophages.

To elucidate the potential mechanisms of PLAU, transcriptomic analysis with three biological replicates was performed on macrophages and macrophages with downregulated PLAU. The gene Ptgs2, which exhibited altered expression following PLAU downregulation and enriched in the NF-κB pathway, was selected for rescue experiments.

### Biological sample acquisition and PBMCs extraction

2 milliliters (ml) fresh peripheral venous blood, diluted with 2 ml phosphate buffered saline (PBS), was collected from LT recipients on preoperative day 1 and postoperative days 1, 3, 5, 7, and 14 using anticoagulant-containing tubes, respectively. 4 ml Ficoll separation solution (Solarbio, P8900) were added to the bottom of SepTube™ centrifuge tube and 4 ml diluted blood were gently added to its surface. After centrifugation (1000g for 30 minutes, room temperature), the fluid was divided into four layers from top to bottom, including plasma, PBMCs, Ficoll separation solution and erythrocytes. PBMCs were aspirated out and the precipitate was taken (1200 rpm for 5 minutes, 4°C) after resuspension with PBS for subsequent experiments. The characteristics of LT patients were listed in **Table 1**.

**Table 1:** Characteristics of LT patients.

Variables	LT patients (n=37)
Age, median (SEM), Years	48.27 (2.02)
Sex, n (%)	
Male	29 (78.38)
Female	8 (21.62)
BMI, median (SEM), kg/m2	23.24 (0.50)
Etiology, n (%)	
Malignant tumor	25 (67.57)
HBV cirrhosis	8 (21.62)
Nodular cirrhosis	1 (2.70)
Primary biliary cirrhosis	2 (5.41)
Drug induced liver failure	1 (2.70)
Cirrhosis, n (%)	
Yes	32 (86.49)
No	5 (13.51)
HBV, n (%)	
Yes	29 (78.38)
No	8 (21.62)
Smoke, n (%)	
Yes	9 (24.32)
No	28 (75.68)
Alcohol use, n (%)	
Yes	5 (13.51)
No	32 (86.49)
Hypertension, n (%)	
Yes	2 (5.41)
No	35 (94.59)
Diabetes, n (%)	
Yes	2 (5.41)
No	35 (94.59)
Surgical procedure, n (%)	
Open	32 (86.49)
Laparoscopy	5 (13.51)
Duration of surgery, median (SEM), minutes	349.2 (10.93)
Blood loss, median (SEM), ml	890.5 (131.3)
Blood transfusion, median (SEM), ml	818.9 (121.2)
ALT on postoperative day 7, median (SEM), U/L	352.1 (22.52)
AST on postoperative day 7, median (SEM), U/L	357.6 (18.15)
Hospital stay, median (SEM), days	25.68 (2.419)

LT, liver transplantation; SEM, standard error mean; BMI, body mass index; HBV, hepatitis B virus; ALT, alanine aminotransferase; AST, aspartate aminotransferase.

### LT model construction and tacrolimus injection

Animal experiments were conducted in the SPF (Specific Pathogen Free) Animal Laboratory of Chongqing Medical University. All experimental rats were 12-week-old male rats weighing 200-220g. Anesthesia was induced using 1% pentobarbital sodium at a dose of 40 mg/kg body weight. Rats in this experiment were euthanized by cervical dislocation. For AR model of LT, the “double-cuff” method (31) was adopted with Lewis rats as donors and Brown Norway (BN) rats as recipients; For sham operation model, BN rats were selected, and the abdominal cavity was closed after exposing the first porta of liver. 8 experimental groups were set up, including Sham, 1d, 3d, 5d, 7d, 14d, 7d+Tac, and 14d+Tac (29), with 6 rats in each group. Of these, rats in 7d+Tac and 14d+Tac were given daily intramuscular Tac (0.5mg/kg body weight) (MCE, FK506) from the first postoperative day until execution. Rats in Sham group were executed on the 1st postoperative day and others were executed on the 1st, 3rd, 5th, 7th and 14th postoperative days, respectively. In each group, 3 rats were used to collect serum and obtain liver tissue, and 3 rats were used to extract liver macrophages.

### *In vivo* transfection

In this study, PLAU-negative control adenovirus (ADV-NC1), PLAU-knockdown adenovirus (ADV-PLAU), Ptgs2-negative control adenovirus (ADV-NC2), and Ptgs2-overexpression adenovirus (ADV-Ptgs2) were purchased from Hanheng Bio. For PLAU transfection, 200 ul of normal saline, ADV-NC1 (1×10^10^ PFU/ml) or ADV-PLAU (1×10^10^ PFU/ml) (29) was injected into the donor liver via the portal vein, and then anastomosed between the donor and recipient portal veins. 4 experimental groups were set up, including Sham, LT, LT+KD-NC and LT+KD, with 12 rats in each group. In Ptgs2 rescue, 200 ul of normal saline, ADV-NC2 (1×10^10^ PFU/ml) or ADV-Ptgs2 (1×10^10^ PFU/ml) (29) was injected into the donor liver via the portal vein of Lewis rats at 14 days prior to surgery, followed by ADV-PLAU transfection during the procedure. 4 groups were set up, including Sham, LT, LT+KD+OE-NC and LT+KD+OE, with 12 rats in each group. In each group, 6 rats were used for survival analysis, 3 were used to obtain serum and liver tissue, and 3 were used to extract liver macrophages for subsequent experiments.

### Liver macrophage extraction and culture

The liver was perfused with saline containing 1% heparin through the portal vein until it was yellow in color, and then with 0.5 mg/ml type IV collagenase (Sigma, C5138) until the surface was gravelly. Chopped the liver, placed in 0.1 mg/ml collagenase type IV and digested at 37°C for 30 minutes. After filtration with a 100 µm filter, cells were washed and resuspended with DMEM complete medium (1200 rpm for 5 min, 4 °C, 3 times). Macrophages were extracted using Percoll solution (Solarbio, P8730) (32). Percoll solution was diluted to 50% and 25% with saline. Successively, 3 ml of 50% and 20% Percoll solution and cell suspension were added into the centrifuge tube, and after centrifugation (1800 g for 30 minutes, 4°C) the solution was divided into 3 layers, from top to bottom, 25% Percoll solution, macrophage and 50% Percoll solution. Macrophages were aspirated, washed three times using PBS (1200 rpm for 5 minutes, 4°C) and cultured using DMEM complete medium (Procell, PM150210B) containing 10% fetal bovine serum (FBS) and 1% penicillin/streptomycin at 37°C and 5% CO_2_.

### Tissue HE, masson, TUNEL, IHC and IF staining

The 4% paraformaldehyde-fixed livers were embedded in paraffin, and then sliced into 5 μm thick sections. Sections were stained with hematoxylin-eosin (HE), TdT-mediated dUTP Nick-End Labeling (TUNEL), Masson, immunohistochemistry (IHC) and immunofluorescence (IF), respectively, by AiFang Biology. Three biological replicates were performed. The dilution concentrations of antibodies used were listed in **Supplementary Table S1**.

### *In vitro* transfection and drug screening

The macrophages used in the vitro experiments were derived from Sprague-Dawley (SD) rats. PLAU-negative control lentivirus (LV-NC1), PLAU-knockdown lentivirus (LV-PLAU), Ptgs2-negative control lentivirus (LV-NC2), and Ptgs2-overexpression lentivirus (LV-Ptgs2) were purchased from Hanheng Bio. *In vitro* lentiviral transfection was performed using the 1/2 small volume infection method according to the instructions. Lentivirus (LV) was added first in 1/2 volume of DMEM complete medium for 4 h before supplementing the other 1/2 of DMEM. According to the instructions, the multiplicity of infection (MOI) of 10, 20 and 30 were set, respectively. The final MOI of LV-NC1 or LV-PLAU with green fluorescence and puromycin (PURO, Biosharp, BS111) resistance, and LV-NC2 or LV-Ptgs2 with blasticidin (BSD, Hanbio, 20240930) resistance, used for subsequent experiments, was verified to be 30. A concentration gradient of 0.5, 1.0, 2.0, 4.0, 6.0, 8.0ug/ml was set for PURO and BSD to treat empty macrophages for 48 h, respectively. The lowest PURO and BSD concentrations that killed more than 90% of the cells were 6ug/ml and 4ug/ml, respectively. 24 h after transfection, PURO or BSD was added for 48 h to screen for successful transfected cells.

### Lipopolysaccharide stimulation

According to the experimental program, macrophages or LV-transfected macrophages were stimulated *in vitro* with 100 ng/ml lipopolysaccharide (LPS) (Biosharp, BS904) for 24 h (29). LPS stimulation could induce macrophage M1-polarization, thereby mimicking the effects of AR.

### Reverse transcription quantitative polymerase chain reaction

According to the instructions, total RNA was extracted using TRIzol reagent (Servicebio, G3013). RNA was reverse transcribed to cDNA using MCE RT kit (HY-K0511). PCR reactions were performed using MCE’s amplification kit, cDNA was used in quantitative Polymerase Chain Reaction (qPCR), using MCE SYBP Green qPCR Master Mix (HY-K0501). Three biological replicates and three technical replicates were performed for reverse transcription quantitative polymerase chain reaction (RT-qPCR). All primers in this study were synthesized by Accurate Biology (AG) and the sequences were shown in **Supplementary Table S2**.

### Western blotting

According to the instructions, total proteins were extracted using RIPA buffer (SparkJade, EA0002), PMSF solution (SparkJade, EA0005) and phosphatase inhibitor (SparkJade, SJ-MK0002) and protein concentrations were detected and leveled using the BCA method (SparkJade, EC0001). After electrophoresis using the PAGE Gel Rapid Preparation Kit (Epizyme Biotech, PG112), proteins were electro-transferred onto polyvinylidene fluoride (PVDF) membranes (Immobilon, IPVH00010). After blocking the PVDF membranes with NcmBlot Blocking Buffer (NCM, P30500) for 10 minutes, the PVDF membranes were placed in primary antibody, and incubated overnight at 60g and 4°C on a shaker. The next day, PVDF membranes were incubated with secondary antibody at room temperature for 1h, and then enhanced chemiluminescence (ECL) was performed using the Oriscience Ultrasensitive ECL Kit (PD202). Three biological replicates were performed. The dilution concentrations of primary and secondary antibodies used in western blotting (WB) were listed in **Supplementary Table S1**.

### Flow cytometry

Each centrifuge tube was filled with 100ul PBS, 1×10^6^ cells and antibodies, and incubated for 1 h at 4 °C in a dark room on a 60g shaker. After incubation, the cells were washed 3 times with PBS, and then resuspended with 100ul PBS for flow cytometry (FCM). In the purity assay, macrophages were stained with CD68-PE antibody before and after purification, respectively. In the M1-polarization ratio assay, macrophages were stained with both CD68-PE and CD86-FITC antibodies. FCM was performed using CytoFLEX and data were analyzed using CytExpert 2.5.0 software (Beckman Coulter). Three biological replicates were performed. The dilution concentrations of the antibodies used in FCM were listed in **Supplementary Table S1**.

### Cellular immunofluorescence

When the density of cells cultured in 3.5 cm glass bottom cell culture dishes (Biosharp, BS-15-GJM) reached 70%, they were fixed by paraformaldehyde (Biosharp, BL539A) for 30 min, permeabilized by immunostaining penetrant with triton X-100 (Beyotime, P0096) for 15 minutes, blocked by goat serum (BOSTER, AR0009) for 1 h at 37 °C, and then incubated overnight at 4 °C with primary antibody. The next day, incubated with secondary antibody for 1 h at room temperature away from light, then added anti-fluorescence quencher with 4’, 6-diamidino-2-phenylindole (DAPI, Beyotime, P0131). IF imaging was performed by the laser confocal microscope (LSM 900 with Airyscan 2). Three biological replicates were performed.The dilution concentrations of the antibodies used in IF were listed in **Supplementary Table S1**.

### Cytokine, ALT and AST detection

Rat blood was allowed to clot naturally for 10 minutes at room temperature and then centrifuged at 3000 rpm for 20 minutes at 4 °C to collect the upper serum layer. Concentrations of IL-1β, IL-6, TNF-α, iNOS, IL-10 and TGF-β were measured by enzyme-linked immunosorbent assay (ELISA) kit (MSKBIO). Concentrations of alanine aminotransferase (ALT) and aspartate aminotransferase (AST) were measured by Micro-ALT Assay Kit (MSKBIO) and Micro-AST Assay Kit (MSKBIO). Three biological replicates were performed.

### Statistical analyses

WB grayscale values were analyzed by Fusion software and the rest of the images were analyzed by Image J software. All data analysis were performed in Graphpad Prism 10.1.2 software. Continuous variables were presented by mean values with standard error mean (SEM), and categorical variables were presented by frequencies with percentages.

For categorical variables, the chi-square test was performed. For continuous variables, the independent samples t-test was used to compare differences between two groups, one-way ANOVA was used to compare differences between multiple groups and Tukey’s honest significant difference (HSD) was used for *post-hoc* test. *P* < 0.05 was considered statistically significant. *, **, ***, and **** denote *P* values less than 0.05, 0.01, 0.001, and 0.0001, respectively.

## Results

### Expression of PLAU and Ptgs2 was increased in AR following clinical LT and rat LT

The results of bioinformatics analysis and transcriptome sequencing were presented in **Supplementary Figure S1**. A total of 1, 071 DEGs between tolerant (Tol) and rejected (NonT) liver tissues were screened out. Among these, 141 core genes (**Supplementary Table S3**) were highly expressed in M1 macrophages and lowly expressed in M2 macrophages. KEGG analysis of these core genes in the bioinformatics revealed that PLAU was enriched in the NF-κB signaling pathway (**Supplementary Table S4**). KEGG analysis of altered genes in the transcriptomics revealed that both PLAU and Ptgs2 were enriched in the NF-κB signaling pathway (**Supplementary Table S5**). Furthermore, following PLAU knockdown, both Ptgs2 and NF-κB expression were downregulated (**Supplementary Table S6**; **Supplementary Figures S1M, N**). NF-κB signaling pathway has been demonstrated to regulate macrophage polarization and AR, but the effects of PLAU/Ptgs2 on AR remains unclear.

**Figure 1:**
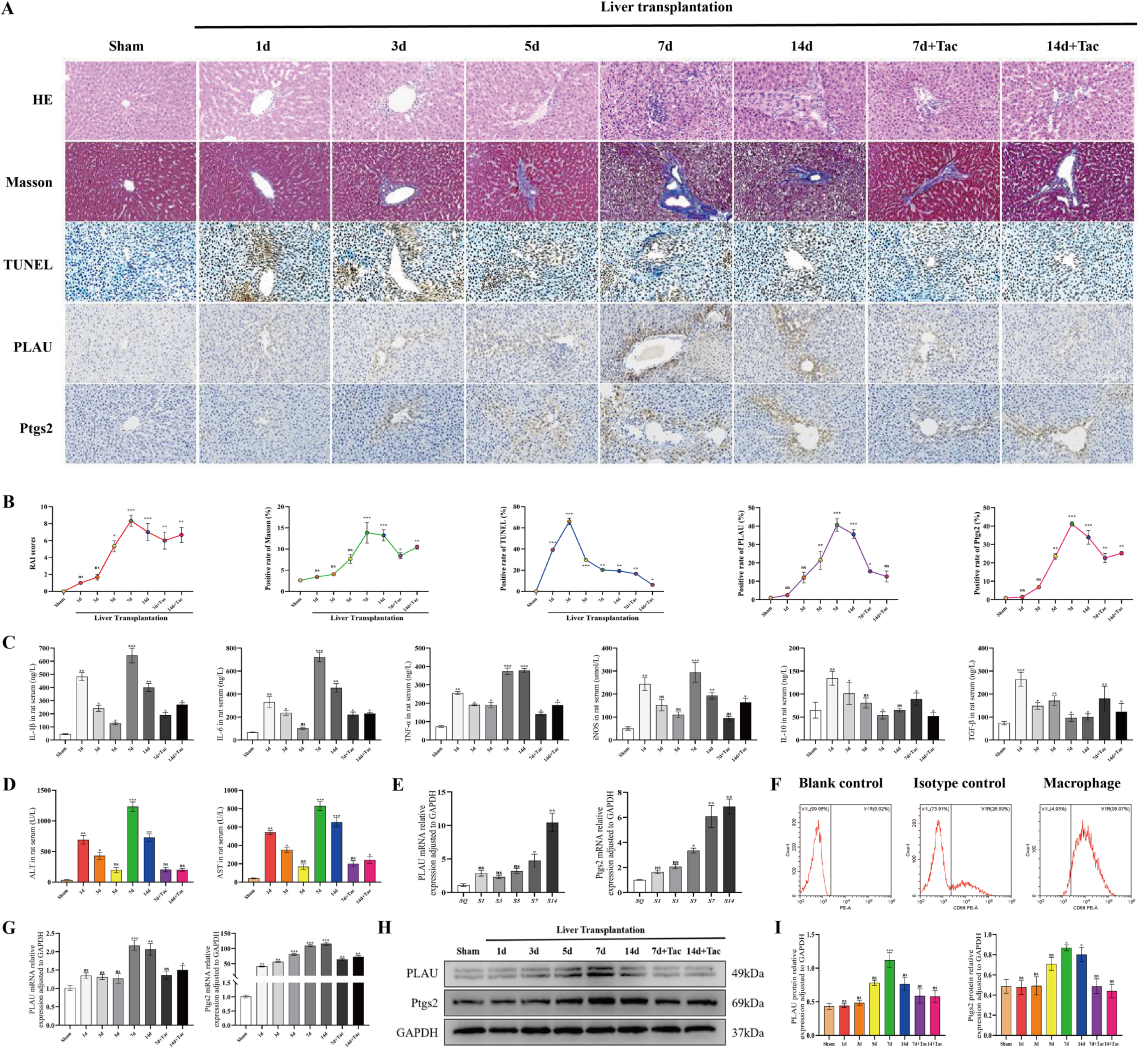
Expression of PLAU and Ptgs2 was increased in AR following clinical LT and rat LT. **(A, B)** Liver tissue HE, Masson, TUNEL and IHC staining and quantitative analysis. **(C, D)** Serum Cytokine, ALT, and AST levels. **(E)** PLAU and Ptgs2 mRNA expression in preoperative and postoperative PBMCs from clinical LT. **(F)** Macrophage purity assessment by FCM. **(G)** PLAU and Ptgs2 mRNA expression in macrophages from rat LT. **(H, I)** PLAU and Ptgs2 proteins expression in rat LT by WB and quantitative analysis. SQ, before surgery. S1, S3, S5, S7, S14, postoperative days 1, 3, 5, 7, and 14. ns, *, **, and *** denote no statistical significance, *P* < 0.05, 0.01, and 0.001, respectively.

To elucidate the dynamic changes in the immune microenvironment following LT, we established a rat LT time-gradient model. We found AR began on the 5th day after LT, and most prominently on the 7th day, accompanied by significant inflammatory cells infiltration, hepatocellular necrosis, and fibrous tissue in the portal area (**Figures 1A, B**), along with markedly elevated proinflammatory cytokines IL-1β, IL-6, TNF-α, and iNOS, and decreased anti-inflammatory cytokines IL-10 and TGF-β (**Figure 1C**), and deteriorated liver function (**Figure 1D**). PLAU and Ptgs2 expression in PBMCs increased after clinical LT and was significantly on postoperative day 7 (**Figure 1E**). Rat liver macrophages were extracted and purified with more than 95% purity (**Figure 1F**), qualifying for experimental use. In rat LT, PLAU and Ptgs2 remained significantly elevated on postoperative day 7 (**Figures 1A, G–I**). However, following tacrolimus treatment, AR was markedly alleviated on postoperative days 7 (7d+Tac) and 14 (14d+Tac), with substantially reduced expression of PLAU and Ptgs2 (**Figures 1A, G–I**). Since AR typically manifested one week after surgery (5), PLAU and Ptgs2 expression was markedly suppressed during the alleviation of AR in rat LT, PLAU and Ptgs2 were suspected to play an important role in AR.

### Low expression of PLAU attenuated AR and reduced macrophage M1-polarization *in vivo*

**Figure 2:**
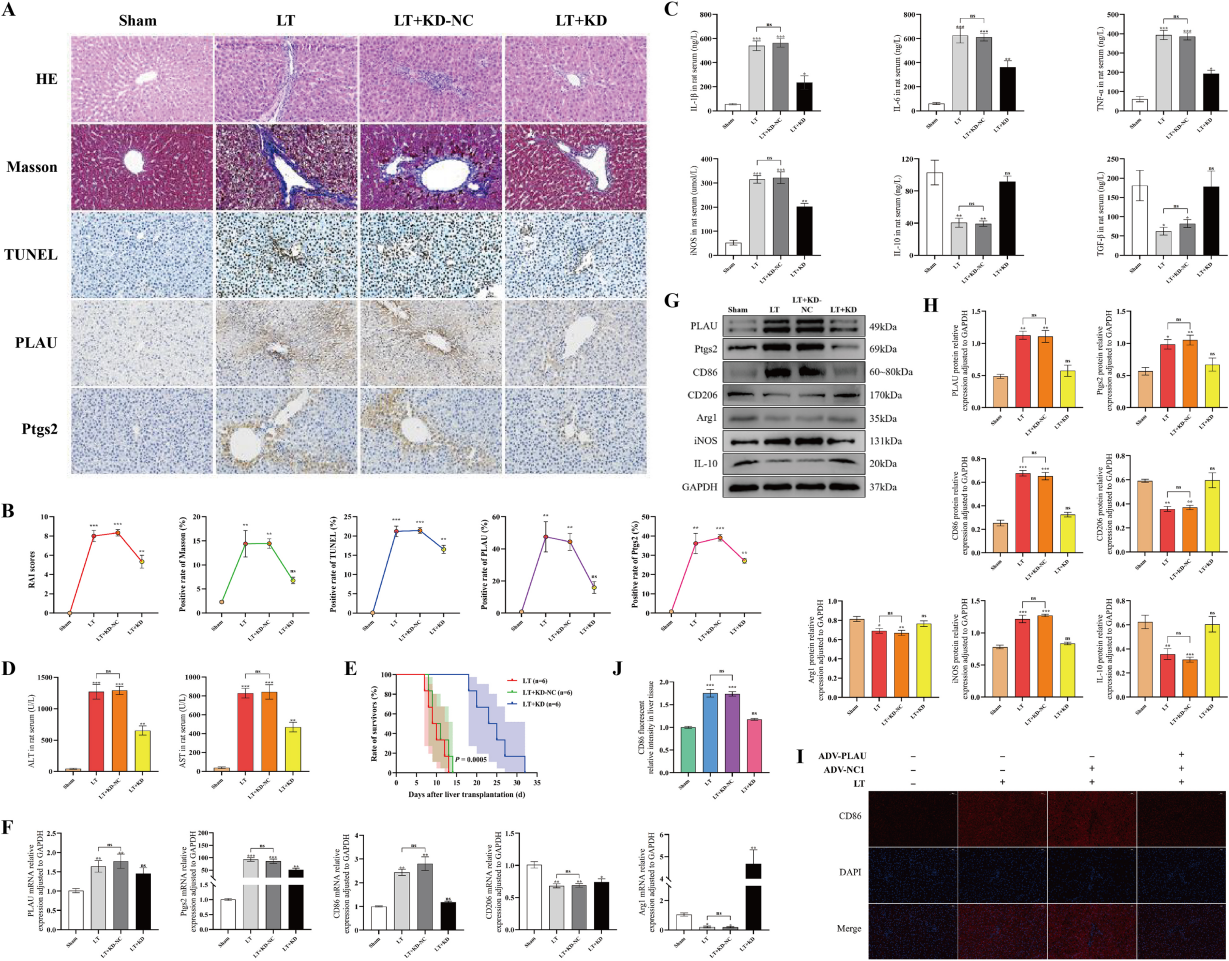
Low expression of PLAU attenuated AR and reduced macrophage M1-polarization *in vivo*. **(A, B)** Liver tissue HE, Masson, TUNEL and IHC staining and quantitative analysis. **(C, D)** Serum Cytokine, ALT, and AST levels. **(E)** Survival curve of LT rats. **(F)** PLAU, Ptgs2 and polarization-associated mRNA expression by RT-qPCR. **(G, H)** PLAU, Ptgs2 and polarization-associated proteins expression by WB and quantitative analysis. **(I, J)** Liver tissue CD86 IF staining and quantitative analysis. ns, *, **, and *** denote no statistical significance, *P* < 0.05, 0.01, and 0.001, respectively.

To clarify the effect of PLAU on AR *in vivo*, we established 4 rat LT groups: Sham, LT, LT+KD-NC, and LT+KD. Compared with LT without knockdown of PLAU, downregulated PLAU exhibited fewer apoptotic hepatocytes, fewer inflammatory cells, less hepatic fibrosis, decreased PLAU and Ptgs2 expression, and a significant amelioration of AR (**Figures 2A, B**). Correspondingly, serum proinflammatory cytokines, ALT and AST decreased, while anti-inflammatory cytokines increased after downregulating PLAU (**Figures 2C, D**). Furthermore, downregulated PLAU (**Figure 2E**).

RT-qPCR and WB performed on macrophages from four groups revealed that, following LT, M1-type macrophage-associated markers CD86 and iNOS increased, while M2-type macrophage-associated markers CD206, Arginase 1 (Arg1), and IL-10 decreased. However, downregulated PLAU resulted in reduced M1-type markers, while promoting the expression of M2-type markers (**Figures 2F–H**). CD86 IF staining of liver tissue consistently supported these findings (**Figures 2I, J**).

### Ptgs2 rescue exacerbated AR and promoted macrophage M1-polarization *in vivo*

**Figure 3:**
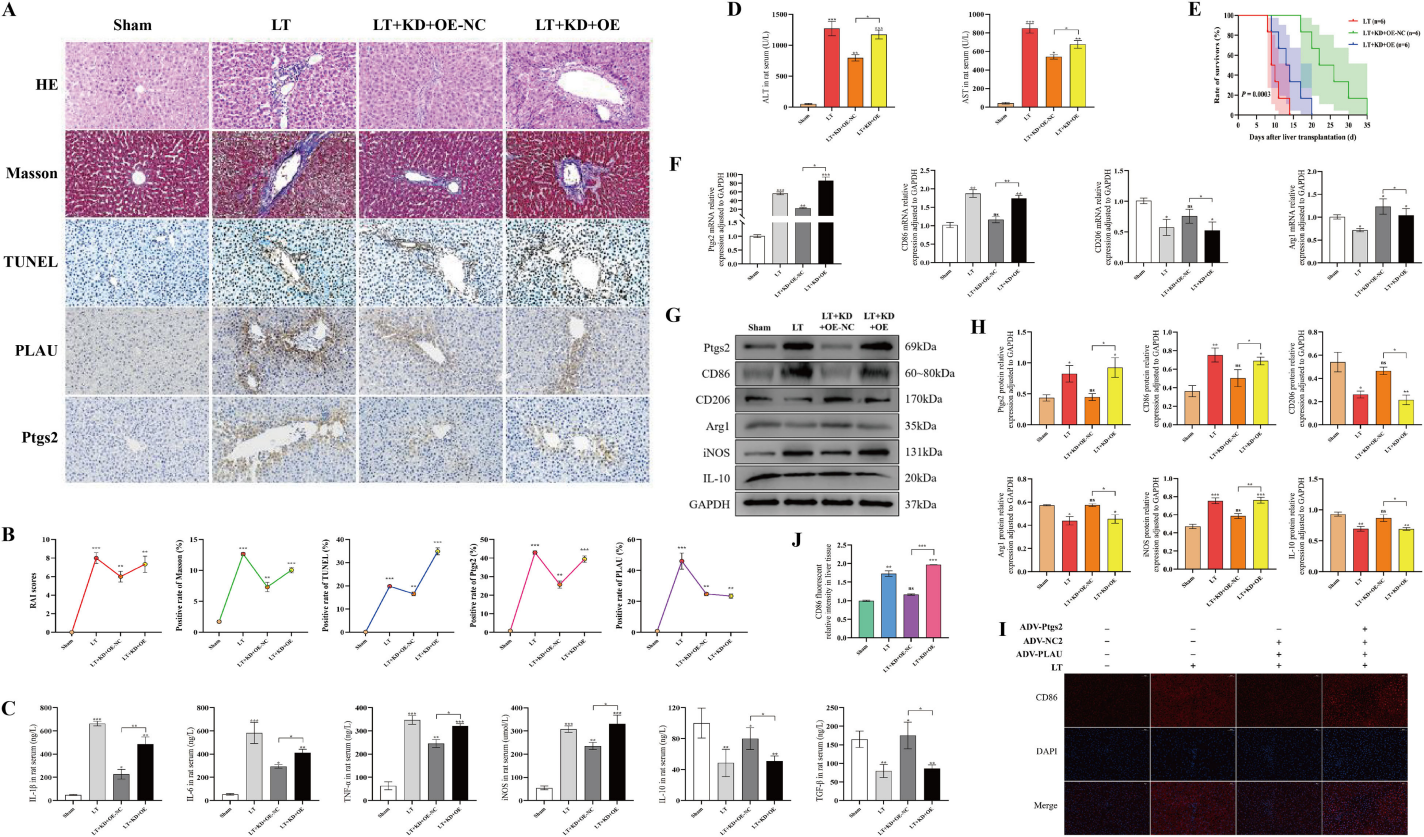
Ptgs2 rescue exacerbated AR and promoted macrophage M1-polarization *in vivo*. **(A, B)** Liver tissue HE, Masson, TUNEL and IHC staining and quantitative analysis. **(C, D)** Serum Cytokine, ALT, and AST levels. **(E)** Survival curve of LT rats. **(F)** Ptgs2 and polarization-associated mRNA expression by RT-qPCR. **(G, H)** Ptgs2 and polarization-associated proteins expression by WB and quantitative analysis. **(I, J)** Liver tissue CD86 IF staining and quantitative analysis. ns, *, **, and *** denote no statistical significance, *P* < 0.05, 0.01, and 0.001, respectively.

To further investigate the mechanism of PLAU, we conducted Ptgs2 rescue experiments. 4 rat LT groups of Sham, LT, LT+KD+OE-NC and LT+KD+OE were established. We found that, as compared to LT rats with downregulated PLAU alone, Ptgs2 rescue exhibited increased hepatocyte apoptosis, enhanced inflammatory cell infiltration, aggravated fibrosis, elevated Ptgs2 expression, and markedly worsened AR (**Figures 3A, B**). Consistently, Ptgs2 rescue also led to elevated serum pro-inflammatory cytokines, ALT, AST, and reduced anti-inflammatory cytokines (**Figures 3C, D**). In addition, Ptgs2 rescue decreased the survival rate of rats (**Figure 3E**).

Similarly, Ptgs2 rescue reversed the suppressive effect of PLAU knockdown on macrophage M1- polarization. PLAU expression showed no significant change, Ptgs2 and M1-polarized macrophages were increased, while M2-polarized macrophages were decreased (**Figures 3A, F–H**). The results of tissue IF also confirmed that Ptgs2 rescue increased CD86 expression (**Figures 3I, J**).

### LPS stimulation promoted PLAU and Ptgs2 expression, induced macrophage M1-polarization *in vitro*

**Figure 4:**
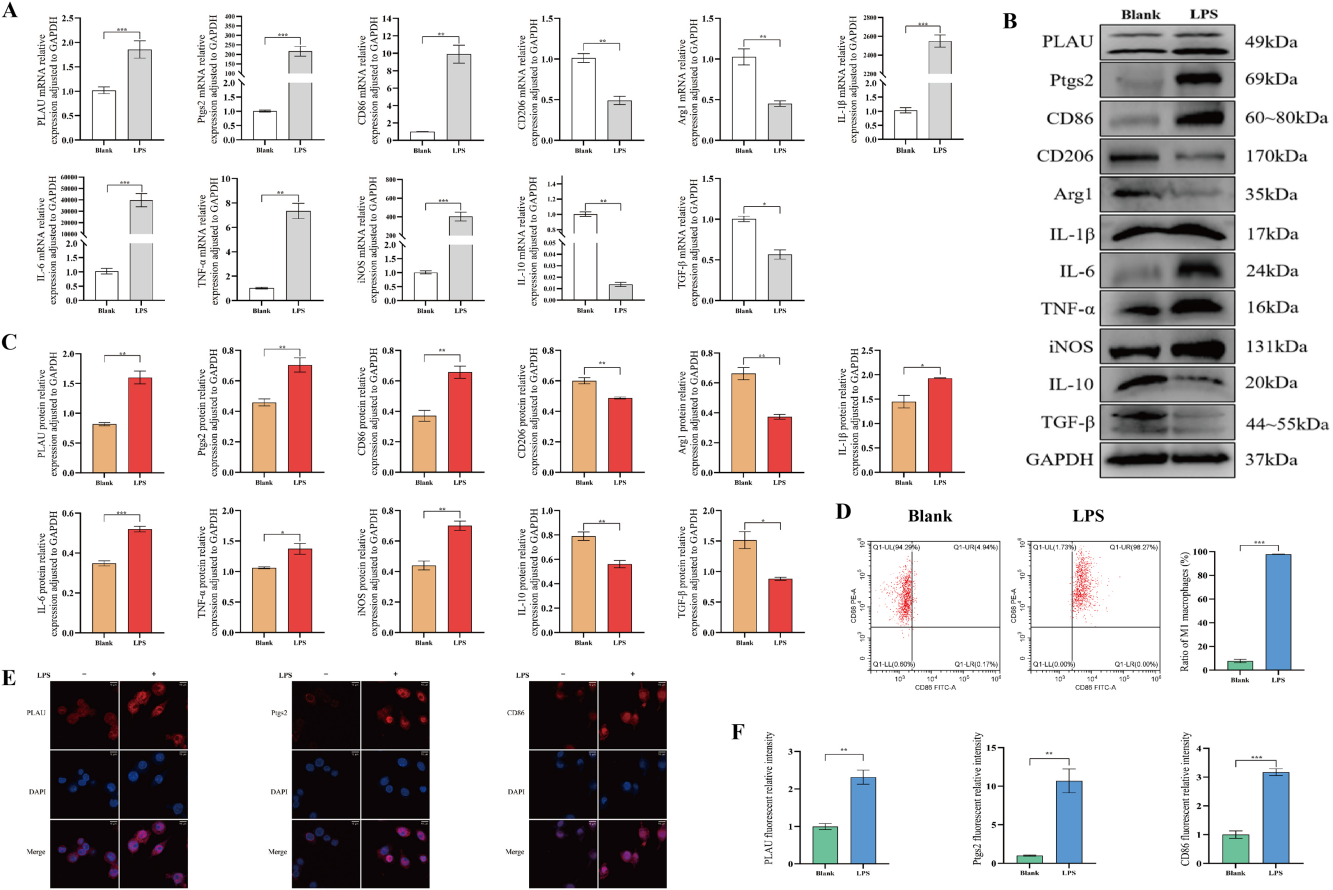
LPS stimulation promoted PLAU and Ptgs2 expression, induced macrophage M1-polarization *in vitro*. **(A)** PLAU, Ptgs2, and polarization-associated mRNA expression by RT-qPCR. **(B, C)** PLAU, Ptgs2, and polarization-associated proteins expression by WB and quantitative analysis. **(D)** Proportion of M1-polarized macrophages by FCM and quantitative analysis. **(E, F)** Macrophages IF staining and quantitative analysis. ns, *, **, and *** denote no statistical significance, *P* < 0.05, 0.01, and 0.001, respectively.

Stimulation of macrophages with LPS increased PLAU and Ptgs2 expression and promoted macrophage M1-polarization. Compared with unstimulated group (Blank), LPS stimulated macrophages exhibited increased M1 polarization-associated markers and decreased M2 polarization-associated markers (**Figures 4A–C**). FCM confirmed over 90% M1-polarization macrophages (**Figure 4D**) and cellular IF showed enhanced expression of PLAU, Ptgs2, and CD86 (**Figures 4E, F**). This indicated that LPS-induced M1 macrophages successfully mimicked AR *in vitro*.

### Low expression of PLAU suppressed macrophage M1-polarization *in vitro*

**Figure 5:**
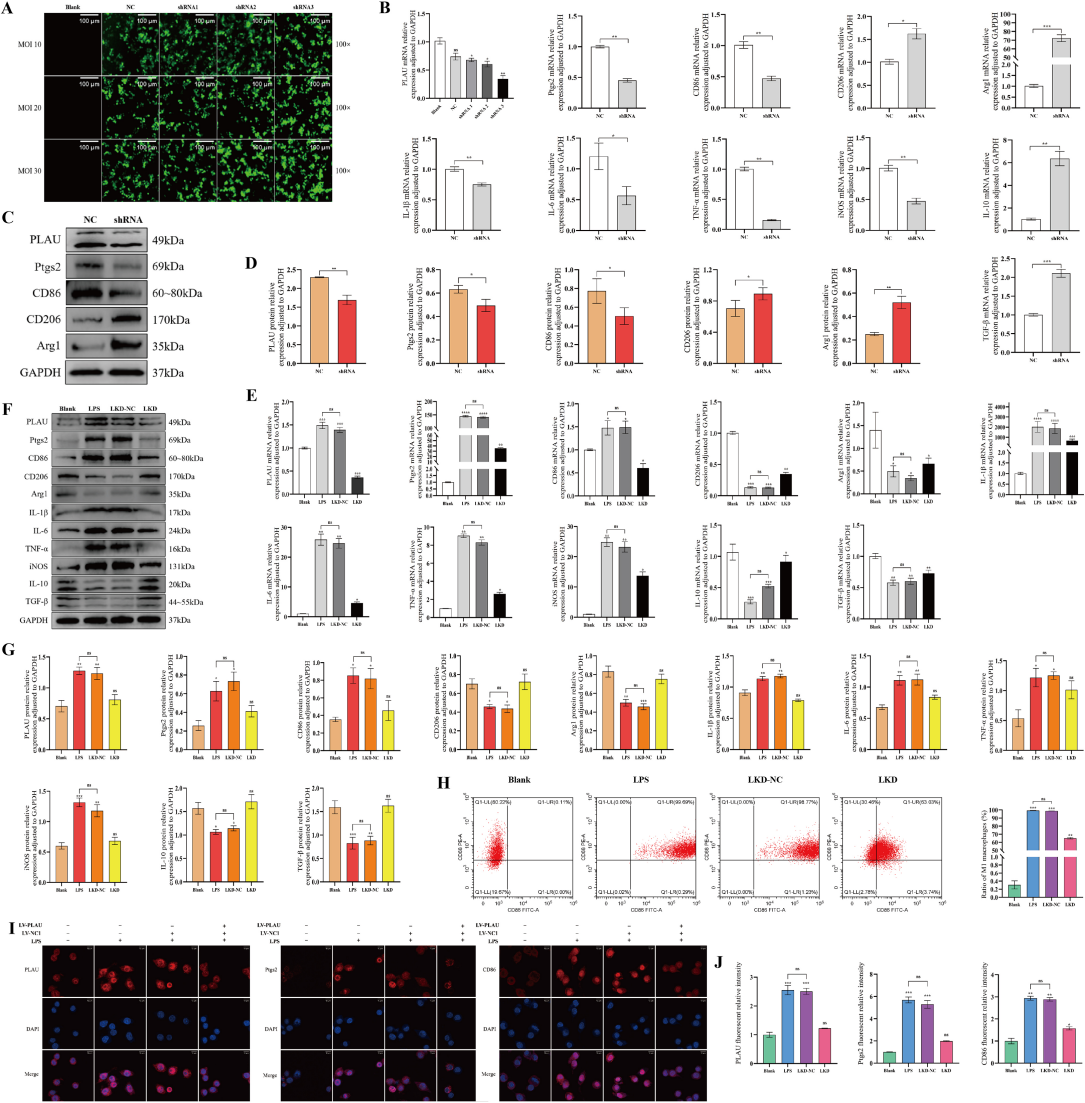
Low expression of PLAU suppressed macrophage M1-polarization *in vitro*. **(A)** Fluorescence intensity of LV transfection. **(B, E)** PLAU, Ptgs2 and polarization-associated mRNA expression by RT-qPCR. **(C, D, F, G)** PLAU, Ptgs2 and polarization-associated proteins expression by WB and quantitative analysis. **(H)** Proportion of M1-polarized macrophages by FCM and quantitative analysis. **(I, J)** Macrophages IF staining and quantitative analysis. ns, *, **, and *** denote no statistical significance, *P* < 0.05, 0.01, and 0.001, respectively.

To clarify the effect of PLAU in macrophage polarization, primary macrophages from SD rats were transfected with LV encoding shRNA1, shRNA2, or shRNA3 to realize PLAU low expression, respectively (**Figure 5A**). Based on optimal fluorescence intensity at MOI 30, shRNA3 demonstrated the highest knockdown efficiency and was selected for subsequent experiments. Even without LPS stimulation, lowly expressed PLAU led to a downward trend in M1-type markers (**Figures 5B–D**).

For further validation, 4 experimental groups, Blank (no intervention), LPS (LPS, 100 ng/mL, 24 h), LKD-NC (LV-NC1 transfection followed by LPS), and LKD (LV-PLAU transfection followed by LPS), were established. Results displayed that PLAU knockdown attenuated LPS-induced macrophage M1-polarization, as evidenced by reduced expression of PLAU, Ptgs2, and M1-type markers, alongside elevated M2-type markers (**Figures 5E–G**). FCM confirmed a significant decrease in M1 macrophage proportion following PLAU knockdown (**Figure 5H**). Similarly, IF showed diminished expression of PLAU, Ptgs2 and CD86 (**Figures 5I, J**). These results collectively demonstrate that PLAU knockdown effectively mitigated LPS-induced M1-polarization in macrophages.

### Ptgs2 rescue promoted macrophage M1-polarization *in vitro*

**Figure 6:**
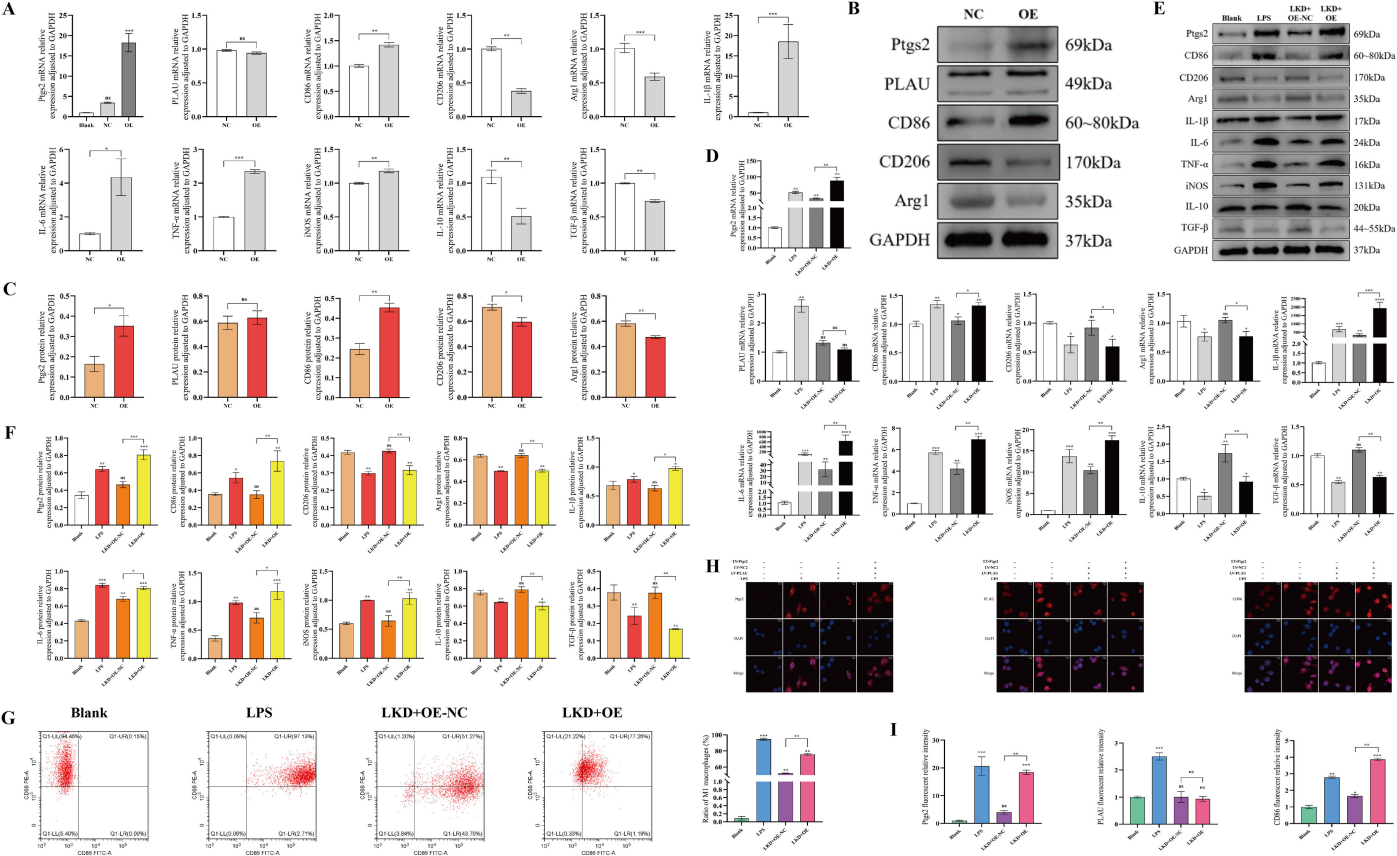
Ptgs2 rescue promoted macrophage M1-polarization *in vitro*. **(A, D)** PLAU, Ptgs2 and polarization-associated mRNA expression by RT-qPCR. **(B, C, E, F)** PLAU, Ptgs2 and polarization-associated proteins expression by WB and quantitative analysis. **(G)** Proportion of M1-polarized macrophages by FCM and quantitative analysis. **(H, I)** Macrophages IF staining and quantitative analysis. ns, *, **, and *** denote no statistical significance, *P* < 0.05, 0.01, and 0.001, respectively.

In the Ptgs2 rescue experiment, we first individually transfected macrophages with Ptgs2 overexpression lentivirus alone to clarify the role of Ptgs2 in macrophages and found that overexpression of Ptgs2 could promote macrophages M1-polarization even without LPS stimulation (**Figures 6A–C**). Subsequently, four macrophage groups were established for Ptgs2 rescue: Blank (no intervention), LPS (LPS, 100 ng/mL, 24 h), LKD+OE-NC (both LV-PLAU and LV-NC2 transfection followed by LPS) and LKD+OE (both LV-PLAU and LV-Ptgs2 transfection followed by LPS). All experimental results indicated that Ptgs2 rescue led to elevated Ptgs2 expression, increased M1-type markers, and decreased M2-type markers (**Figures 6D–I**), indicating Ptgs2 rescue reversed the suppression of M1-polarization mediated by downregulating PLAU.

### LPS stimulation activated AKT/NF-κB pathway in macrophage *in vitro*

**Figure 7:**
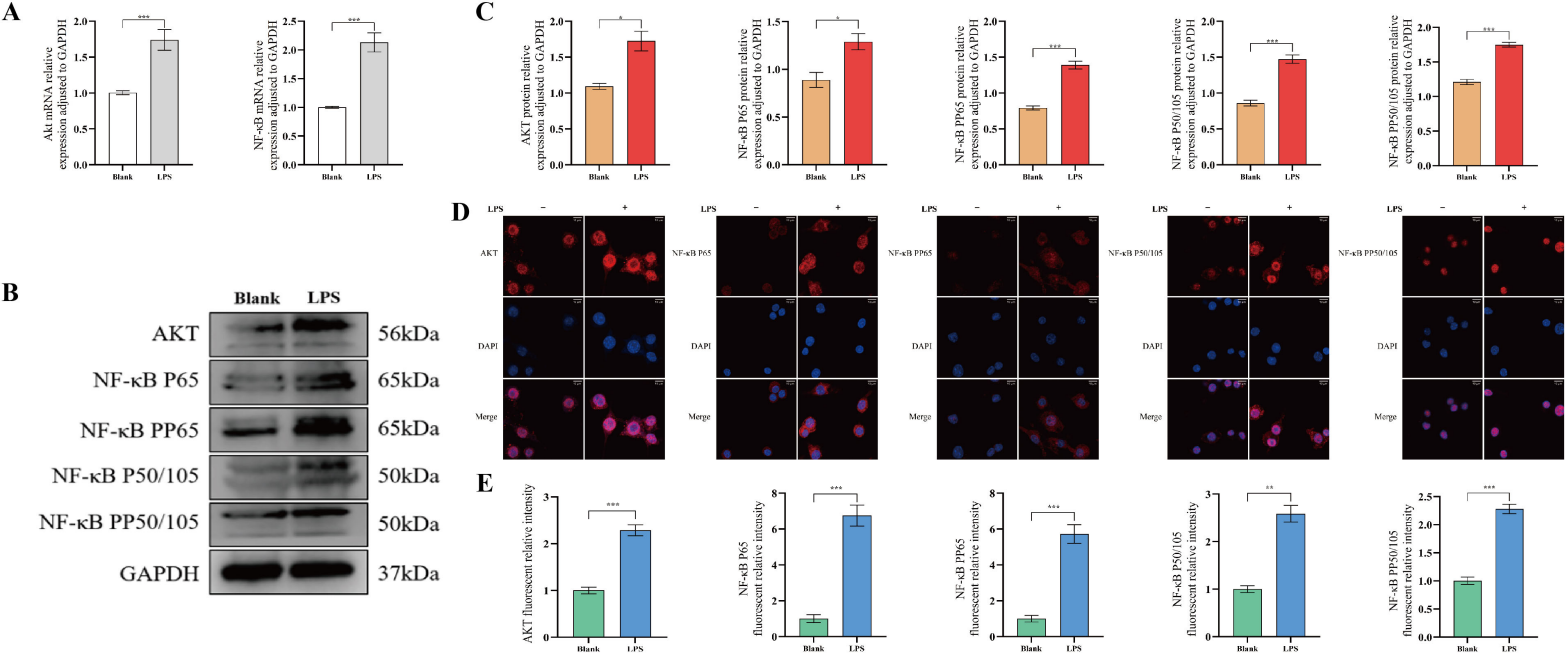
LPS stimulation activated the AKT/NF-κB pathway in macrophages *in vitro*. **(A)** AKT/NF-κB pathway mRNA expression by RT-qPCR. **(B, C)** AKT/NF-κB pathway protein expression by WB and quantitative analysis. **(D, E)** Macrophages IF staining and quantitative analysis. ns, *, **, and *** denote no statistical significance, *P* < 0.05, 0.01, and 0.001, respectively.

To explore the potential mechanisms of the PLAU/Ptgs2 axis, KEGG enrichment analysis in bioinformatics and transcriptomics analyses revealed that both PLAU and Ptgs2 were enriched in NF-κB pathway (**Supplementary Figures S1M, N**; **Supplementary Tables S4**–**S6**). Previous studies had demonstrated that AKT regulated NF-κB in macrophages (15), suggesting that the AKT/NF-κB pathway might serve as a potential signaling pathway for the PLAU/Ptgs2 axis. LPS stimulation confirmed significant activation of the AKT/NF-κB pathway in macrophages, with elevated expression of AKT, nuclear factor kappa B p65 (NF-κB P65), phospho-NF-κB p65 (NF-κB PP65), NF-κB P50/105, and phospho- NF-κB P50/105 (NF-κB PP50/105) (**Figures 7A–E**).

### Low expression of PLAU inhibited the AKT/NF-κB pathway in macrophages *in vitro*

**Figure 8:**
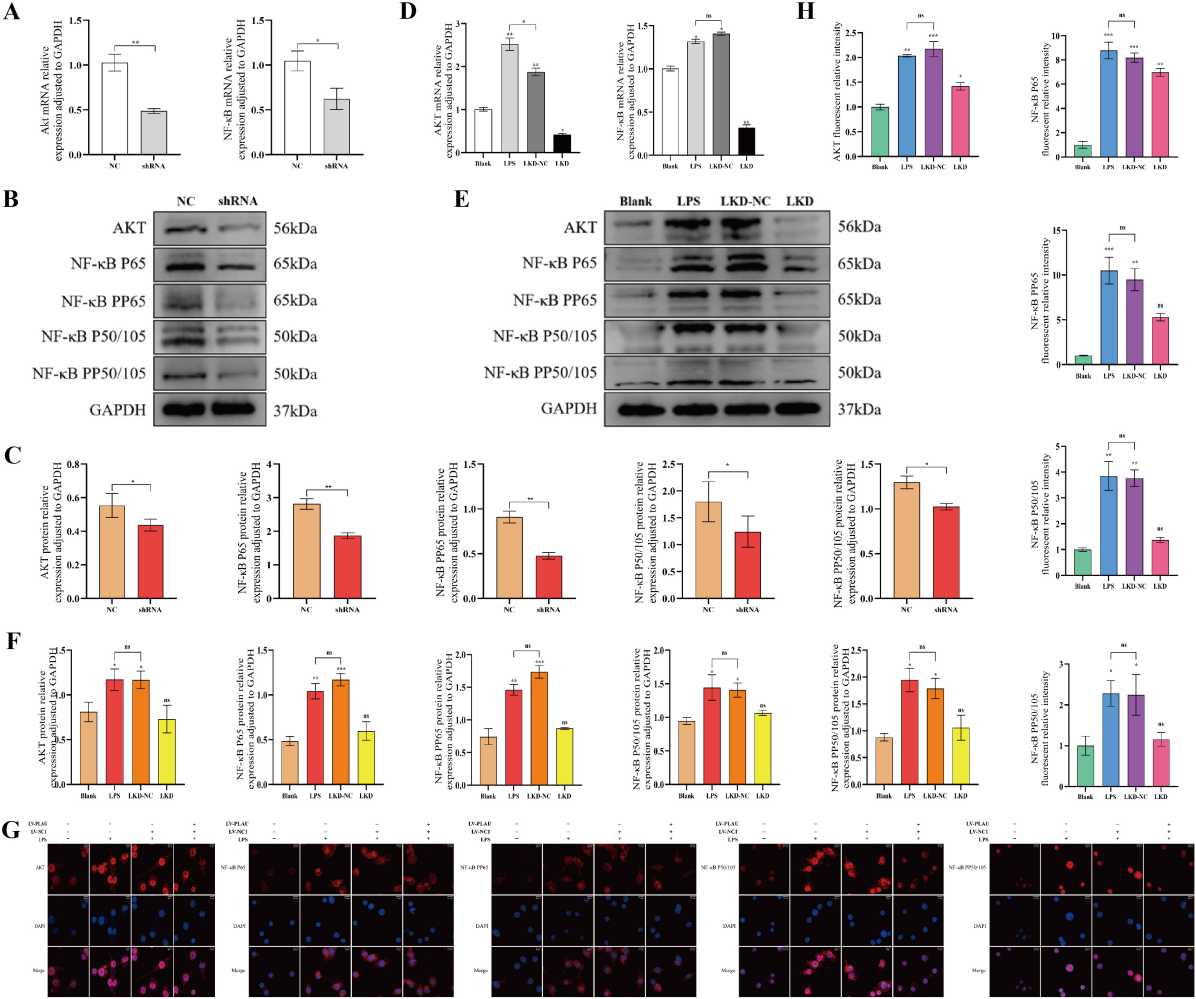
Low expression of PLAU inhibited the AKT/NF-κB pathway in macrophages *in vitro*. **(A, D)** AKT/NF-κB pathway mRNA expression by RT-qPCR. **(B, C, E, F)** AKT/NF-κB pathway proteins expression by WB and quantitative analysis. **(G, H)** Macrophages IF staining and quantitative analysis. ns, *, **, and *** denote no statistical significance, *P* < 0.05, 0.01, and 0.001, respectively.

To determine whether the AKT/NF-κB pathway was regulated by PLAU/Ptgs2 axis, we interfered with PLAU expression in macrophages. Even prior to LPS stimulation, downregulated PLAU inhibited AKT/NF-κB pathway expression in macrophages (**Figures 8A–C**). Following LPS stimulation, pathway activity was substantially inhibited in the LKD group, with the transcriptional and translational levels of AKT, NF-κB P65, PP65, P50/105, PP50/105 were restricted (**Figures 8D–F**). IF analysis confirmed markedly diminished intensity of these signaling components (**Figures 8G, H**), collectively demonstrating the potent inhibitory effect on AKT/NF-κB signaling by low expression of PLAU.

### Ptgs2 rescue activated AKT/NF-κB pathway in macrophages *in vitro*

**Figure 9:**
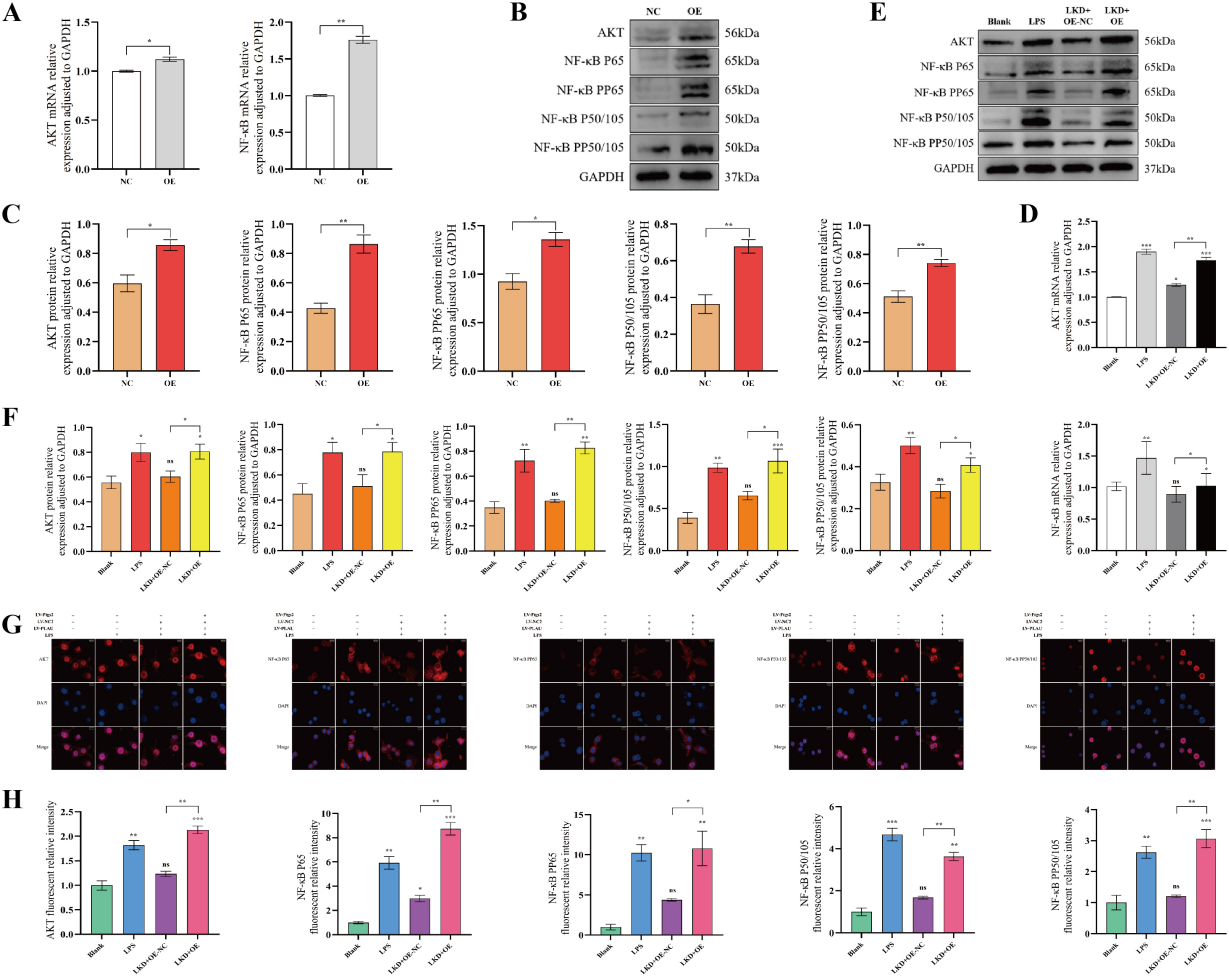
Ptgs2 rescue activated AKT/NF-κB pathway in macrophages *in vitro*. **(A, D)** AKT/NF-κB pathway mRNA expression by RT-qPCR. **(B, C, E, F)** AKT/NF-κB pathway proteins expression by WB and quantitative analysis. **(G, H)** Macrophages IF staining and quantitative analysis. ns, *, **, and *** denote no statistical significance, *P* < 0.05, 0.01, and 0.001, respectively.

To further confirm the regulation of the AKT/NF-κB pathway by PLAU/Ptgs2, we performed Ptgs2 rescue experiments. Overexpression of Ptgs2 in macrophages resulted enhanced the expression of AKT, NF-κB P65, PP65, P50/105, PP50/105 (**Figures 9A–C**). After LV transfection and LPS stimulation, results suggested that the AKT/NF-κB pathway remained more active in the Ptgs2 rescue groups (**Figures 9D–H**). Therefore, Ptgs2 rescue reversed the inhibitory effect of PLAU knockdown on AKT/NF-κB pathway in macrophages, both with and without LPS stimulation.

## Discussion

Liver transplantation (LT) as a treatment for end-stage liver disease faced significant challenges, including acute rejection (AR) and adverse effects associated with long-term immunosuppressive therapy (33–35). Macrophage M1-polarization was closely linked to AR, while M2-polarization promoted immune tolerance (36). Identifying new targets that modulated macrophage polarization might provide new strategies for mitigating AR and improving clinical outcomes.

In this study, we revealed that AR in LT significantly occurred on the 7th postoperative day, PLAU and Ptgs2 were significantly increased in both clinical LT patients and in LT rats on day 7 post-surgery, consistent with clinical timing of AR (5). Furthermore, following tacrolimus treatment, the expression of PLAU and Ptgs2 decreased significantly as AR alleviated. Therefore, PLAU and Ptgs2 were highly suspected to be involved in the regulation of AR. Downregulated PLAU led to a decrease of Ptgs2 expression, proinflammatory cytokines, and macrophage M1-polarization, thereby attenuating AR. Ptgs2 rescue increased proinflammatory responses and macrophage M1-polarization, exacerbating AR but without affecting PLAU expression significantly, suggesting that PLAU/Ptgs2 axis might regulate AR by modulating macrophage polarization. Additionally, to clarify the potential mechanisms of PLAU/Ptgs2 axis, we stimulated macrophages with LPS to induce M1 polarization, thereby mimicking AR *in vitro* (37), and identified AKT/NF-κB as a potential signaling pathway regulating macrophage polarization. Consequently, the PLAU/Ptgs2 axis might guide the clinical prevention and treatment of AR. For instance, tacrolimus might alleviate AR by inhibiting the PLAU/Ptgs2 axis, thereby modulating the AKT/NF-κB pathway and suppressing macrophage M1-polarization. This provided a new perspective on the pleiotropic effects of immunosuppressants in clinical practice, extending their action beyond classical T-cell targeting to include regulation of innate immunity. These findings also provided a theoretical basis for future combination therapies targeting the PLAU/Ptgs2 axis.

PLAU and Ptgs2 were widely and lowly expressed in a variety of cells in physiological states, while significant changed under inflammation, injury and cancer (38, 39). PLAU was lowly expressed in myocardial infarction (17), AD (40), and fibrotic diseases (13); but highly expressed in cancer (41, 42) and hemorrhagic disorders (43). Elevation of PLAU was a risk factor for hemorrhage after LT (20). Ptgs2 was overexpressed in many diseases, including cancer (44), neurodegenerative diseases (45), ischemic stroke (46), and leukemia (47). It had been demonstrated that Ptgs2 overexpression leaded to exacerbation of I/RI after LT, whereas inhibition of COX-2 could improve liver function (25). Studies had confirmed that the AKT/NF-κB pathway participated in macrophage polarization in various diseases (26–28). However, studies on AR in LT had mainly focused on NF-κB, with relatively few studies examining the AKT/NF-κB pathway (29, 30). Our research revealed that the downregulated PLAU/Ptgs2 axis inhibited M1-polarization through the AKT/NF-κB pathway, thereby improving AR.

Although the AKT/NF-κB pathway was considered a core hub in macrophage polarization, its upstream regulatory mechanisms in acute rejection of liver transplantation remained unclear. This study revealed that PLAU and Ptgs2 might specifically activate this pathway through synergistic effects. In the rejection microenvironment, inflammatory factors stimulated macrophages to highly express PLAU, whose receptor uPAR could initiate signals such as integrin, thereby activating the AKT/NF-κB pathway (14). Simultaneously, inflammatory signals induced high expression of Ptgs2, whose product PGE2 activated the cAMP-PKA pathway via G protein-coupled receptors. PKA directly phosphorylated AKT while also enhancing AKT signaling by inhibiting PTEN (a negative regulator of AKT) (48). Activated AKT subsequently phosphorylated IKKα/β, NF-κB p50/105, and the NF-κB p65 subunit (Ser536) (49), promoting nuclear translocation of NF-κB and amplifying its transcriptional activity, thereby establishing sustained inflammatory signaling. This study confirmed that downregulated PLAU inhibited the AKT/NF-κB pathway and its phosphorylation, while overexpressed Ptgs2 exerted the opposite effect. These findings revealed a new mechanism whereby the PLAU/Ptgs2 axis, acting as an upstream regulator, drove M1-polarization of macrophages through a cascade amplification mechanism in liver transplant rejection.

There were several limitations in this study. Firstly, our study utilized only PBMCs from clinical LT patients due to limited availability of liver tissue. Due to distinct microenvironment inside and outside the liver, the PLAU/Ptgs2 axis might function differently, promoting M1-polarization of PBMCs in the periphery while regulating M1/M2 balance in hepatic macrophages. However, monocytes within PBMCs were recruited to the liver and differentiated into macrophages during AR. Therefore, it was reasonable to monitor PBMCs to reflect AR in liver. Secondly, routinely anti-rejection treatment resulted in a lack of symptomatic AR, thereby preventing stratified analysis of LT patients based on the presence or absence of AR. However, genetic changes occurred earlier than phenotypic changes. PLAU and Ptgs2 expression peaked on postoperative day 7 and decreased significantly following immunosuppressive therapy, potentially reflecting their involvement in AR. Finally, macrophage depletion was not performed in rats because it was difficult to determine whether remission of AR was merely due to changes in liver macrophages rather than those from other organs.

## Data Availability

The original contributions presented in the study are included in the article/**Supplementary Material**. Further inquiries can be directed to the corresponding authors.
