# Supplementary material for: Downregulated PLAU alleviates acute rejection after liver transplantation by targeting Ptgs2 in macrophages
Source: Front Immunol. 2026 Mar 31;17:1779520. doi: 10.3389/fimmu.2026.1779520 (PMC13078257; doi:10.3389/fimmu.2026.1779520)
Supplement: Supplementary file 1 [file DataSheet1.pdf]

Supplementary Material

Supplementary Figures and Tables

1.1 Supplementary Figures

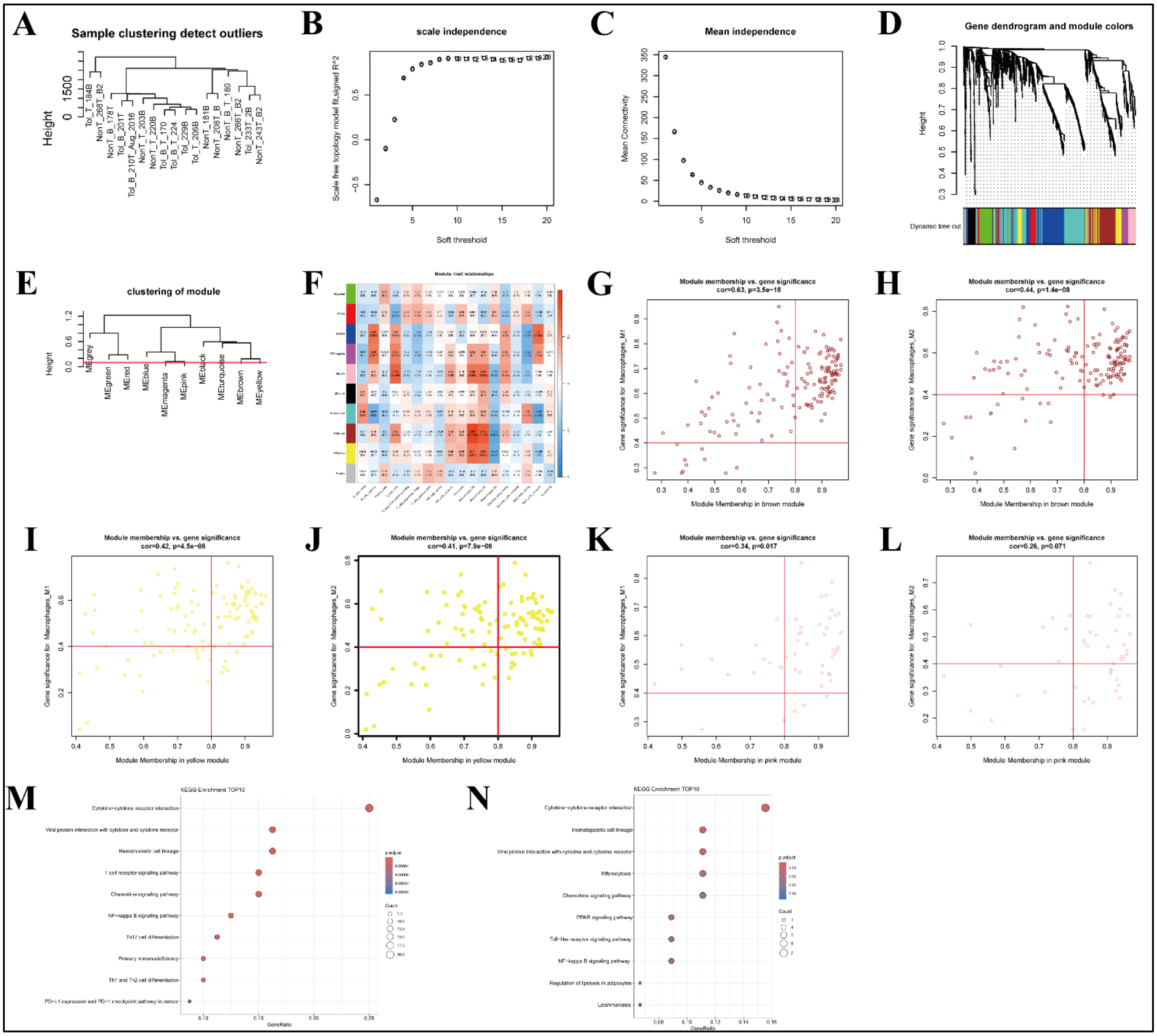

Fig. S1 Bioinformatics analysis and transcriptome sequencing. (A) Sample tree across 17 LT cases. (B-C) Determination of soft threshold $\beta = 9$ through scale-free topology analysis. (D-E) Hierarchical clustering dendrogram with 10 co-expression modules (color-coded). (F) Module-lymphocyte pathway correlation heatmap. (G-L) 141 core genes derived from the brown, pink, and yellow modules with trait-relatedness > 0.4 and module membership > 0.8. (M) KEGG functional enrichment analysis bubble chart of core genes in clinical LT cases. (N) KEGG functional enrichment analysis bubble chart of genes in macrophages with low PLAU expression.

1.2 Supplementary Tables

Table S1. Antibody dilution concentrations for WB, FCM, IF and IHC.

Antibody	Supplier	Catalog Number	Dilution
GAPDH	Immunoway	YM8016	WB: 1:1000
PLAU	Huabio	ET1703-26	WB: 1:1000; IF: 1:200; IHC: 1:100
HIF1α	Zenbio	340462	WB: 1:1000; IF: 1:200
Ptgs2	Huabio	ET1610-23	WB: 1:1000; IF: 1:200; IHC: 1:100
CD86	Zenbio	R380350	WB: 1:1000; IF: 1:200
CD206	Immunoway	YT5640	WB: 1:1000
Arg1	Immunoway	YT0311	WB: 1:1000
AKT	Immunoway	YT0185	WB: 1:1000; IF: 1:200
NF-κB P65	Immunoway	YT3108	WB: 1:1000; IF: 1:200
NF-κB PP65	Zenbio	310013	WB: 1:1000; IF: 1:200
NF-κB P50/105	Immunoway	YT3101	WB: 1:1000; IF: 1:200
NF-κB PP50/105	Zenbio	310171	WB: 1:1000; IF: 1:200

IL-1β	Immunoway	YT5201	WB: 1:1000
IL-6	Proteintech	26404-1-AP	WB: 1:1000
TNF-α	Immunoway	YT4689	WB: 1:1000
iNOS	Immunoway	YT3169	WB: 1:1000
IL-10	Immunoway	YT5138	WB: 1:1000
TGF-β	Immunoway	YT4632	WB: 1:1000
HRP* Goat Anti-Rabbit IgG (H+L)	Immunoway	RS0002	WB: 1:5000
HRP* Goat Anti-Mouse IgG (H+L)	Immunoway	RS0001	WB: 1:5000
CD68-PE	eBioscience	2558786	FCM: 1:100
CD86-FITC	eBioscience	2691707	FCM: 1:100
Cy3−conjugated Goat Anti-Rabbit IgG (H+L)	Beyotime	A0516	IF: 1:200

WB, western blotting. FCM, flow cytometry. IF, Immunofluorescence. IHC, immunohistochemistry. GAPDH, glyceraldehyde-3-phosphate dehydrogenase. PLAU, urokinase-type plasminogen activator. HIF1α, hypoxia-inducible factor alpha. Ptgs2, prostaglandin-endoperoxide synthase 2. CD, cluster of differentiation. Arg1, arginase1. AKT, protein kinase B. NF-κB, nuclear factor kappa B.

Table S2. Sequences of primers.

Primers	Sequences	
Homo-GAPDH	F:	GCACCGTCAAGGCTGAGAAC
	R:	TGGTGAAGACGCCAGTGGA
Homo-PLAU	F:	AGGCTTAACTCCAACACGCA
	R:	ACGGATCTTCAGCAAGGCAA
Homo-Ptgs2	F:	TGATGATTGCCCGACTCCCT
	R:	TGGCCCTCGCTTATGATCTGT
Rno-GAPDH	F:	GGTGGACCTCATGGCCTACA
	R:	CTCTCTTGCTCTCAGTATCCTTGCT
Rno-PLAU	F:	GCAAGCAGCCTCACTACTATGG
	R:	GGTCCTCCTGAATCTCCCGA
Rno-HIF1α	F:	GCTTCCTTCGATCAGTTGTC
	R:	TCATCAGTGGTGGCAGTAGC
Rno-Ptgs2	F:	TTCGGGAGCACAACAGAGTGT
	R:	GAGCCTGCTGGTTTGGAACAG
Rno-CD86	F:	CCTGCTGGCTGTGTTTGTT
	R:	AGGCGGTAGGTGTAGGTGA
Rno-CD206	F:	GGACCTTTGGATGGCTACCA
	R:	TCCAGGTCCTTGTTGAGC AT
Rno-Arg1	F:	CCGCAGCATTAAGGAAAGC

	R:	CCCGTGGTCTCTCACATTG
Rno-AKT	F:	CGCTTCTTTGCCAACATCGT
	R:	GTGTCGGTCTCAGAGGTGAC
Rno-NF-κB	F:	CTGCCGAGTAAACCGGAACT
	R:	GCCAGGTCCCGTGAAATACA
Rno-IL-1β	F:	AATCTCACAGCAGCATCTCGACAAG
	R:	TCCACGGGCAAGACATAGGTAGC
Rno-IL-6	F:	ACTTCCAGCCAGTTGCCTTCTTG
	R:	TGGTCTGTTGTGGGTGGTATCCTC
Rno-TNF-α	F:	AAAGGACACCATGAGCACGGAAAG
	R:	CGCCACGAGCAGGAATGAGAAG
Rno-iNOS	F:	TTTGGCCCGAAGGTCGC
	R:	AGGGATTCTGGAACATTCTGTG
Rno-IL-10	F:	TGCTATGTTGCCTGCTCTTACTG
	R:	TCAAATGCTCCTTGATTTCTGG
Rno-TGF-β	F:	TTGCTTCAGCTCCACAGAGA
	R:	TGGTTGTAGAGGGCAAGGAC

GAPDH, glyceraldehyde-3-phosphate dehydrogenase. PLAU, urokinase-type plasminogen activator. HIF1α, hypoxia-inducible factor alpha. Ptgs2, prostaglandin-endoperoxide synthase 2. CD, cluster of differentiation. Arg1, arginase1. AKT, protein kinase B. NF-κB, nuclear factor kappa B.

Table S3. 141 core genes with trait-relatedness greater than 0.4 and module membership greater than 0.8 in bioinformatics.

Gene	M1 macrophages	M2 macrophages	Mebrown	MEyellow	MEpink
ABCD2	0.5429	-0.4669	0.8171	0.7258	0.6042
ACAP1	0.7875	-0.6581	0.8168	0.4922	0.9406
AL365361.1	0.6566	-0.5866	0.8915	0.8490	0.5961
ANKRD44	0.5085	-0.4861	0.6536	0.9113	0.2616
APOL4	0.7185	-0.5170	0.8494	0.6638	0.7008
ARHGAP30	0.5942	-0.5412	0.8131	0.9008	0.5519
ASB2	0.6609	-0.5117	0.9086	0.7631	0.7684
BCL11B	0.6762	-0.5773	0.9473	0.7415	0.7607
BCL2A1	0.5499	-0.4258	0.8621	0.5137	0.9434
BTG2	0.6236	-0.6456	0.8774	0.9051	0.6173
BTLA	0.7731	-0.6517	0.9283	0.7356	0.7855
CARD11	0.6817	-0.6217	0.8755	0.9085	0.5822
CCL18	0.7713	-0.6109	0.9211	0.6143	0.8764
CCL4	0.6378	-0.4630	0.6431	0.2437	0.9025
CCNG2	0.5316	-0.5147	0.6852	0.8553	0.4377
CCR4	0.7051	-0.6199	0.9269	0.7528	0.7111
CCR5	0.6120	-0.5255	0.9409	0.8323	0.7257

CCR7	0.6995	-0.5512	0.9004	0.5842	0.8347
CCRL2	0.5912	-0.4805	0.8912	0.6121	0.8593
CD1E	0.7885	-0.6334	0.8123	0.4205	0.8445
CD2	0.6917	-0.5814	0.9560	0.7027	0.8641
CD28	0.6194	-0.5251	0.9104	0.7813	0.6398
CD37	0.6261	-0.5223	0.6778	0.2506	0.9248
CD3D	0.5371	-0.4200	0.7654	0.3537	0.9482
CD3E	0.7267	-0.5968	0.9379	0.7158	0.7993
CD3G	0.6770	-0.6266	0.9383	0.8226	0.7236
CD5	0.6068	-0.4806	0.9329	0.7172	0.7468
CD6	0.6063	-0.4855	0.9228	0.7064	0.7530
CD69	0.7536	-0.6165	0.9259	0.7609	0.7464
CD83	0.6899	-0.5492	0.9446	0.7912	0.7162
CD8B	0.8538	-0.7732	0.8123	0.5935	0.8497
CD96	0.7422	-0.6347	0.9596	0.8038	0.7455
CELF2	0.6597	-0.6228	0.9016	0.8897	0.6235
CHD3	0.6631	-0.6901	0.6802	0.8162	0.2677
CHST2	0.6488	-0.5211	0.8915	0.7560	0.6603
CIITA	0.6355	-0.5169	0.8974	0.9287	0.6219
CLEC2D	0.7369	-0.6212	0.8528	0.5645	0.9096
CSF2RB	0.5191	-0.4626	0.8223	0.8694	0.5375

CXCL10	0.7721	-0.6772	0.9453	0.7390	0.7753
CXCL11	0.7506	-0.6380	0.9583	0.8228	0.7111
CXCL9	0.8170	-0.7048	0.9704	0.8099	0.7351
CXCR3	0.6890	-0.5770	0.9262	0.6431	0.8131
CXCR4	0.6875	-0.6024	0.8823	0.5359	0.9390
CYTIP	0.6070	-0.4672	0.7730	0.4505	0.8416
DEF6	0.6285	-0.5754	0.7479	0.4827	0.8494
DOCK10	0.4555	-0.4351	0.6904	0.9216	0.3165
FLNA	0.5411	-0.5648	0.7557	0.9232	0.3684
FLT3	0.6385	-0.4733	0.8450	0.6847	0.6825
FMN1	0.2866	-0.2574	0.4691	0.8319	0.1253
FMNL3	0.5296	-0.4126	0.7686	0.9253	0.3921
FSTL3	0.6483	-0.6026	0.8342	0.6411	0.7482
GAB3	0.6438	-0.4904	0.9091	0.8501	0.7319
GAS7	0.5845	-0.5216	0.7717	0.9453	0.3912
GBP2	0.6833	-0.5458	0.9354	0.7981	0.7589
GBP5	0.5889	-0.4957	0.9215	0.8335	0.6520
GLIPR2	0.6634	-0.5525	0.9132	0.6529	0.8574
GP1BA	0.6789	-0.6419	0.8459	0.7811	0.5502
GPNMB	0.6385	-0.5819	0.9379	0.7949	0.6790

GPR171	0.7587	-0.5809	0.8902	0.5575	0.8864
GPR174	0.7065	-0.6192	0.8993	0.8264	0.6451
GPRIN3	0.5842	-0.4666	0.7277	0.8608	0.3931
GRAMD1B	0.7030	-0.6426	0.8552	0.8630	0.5792
GRAP2	0.6823	-0.5679	0.9507	0.7810	0.7470
GZMA	0.6035	-0.4020	0.8745	0.5616	0.9184
HAPLN3	0.6262	-0.5431	0.9252	0.6493	0.8771
HOPX	0.6718	-0.4711	0.7719	0.3738	0.9496
IDO1	0.7140	-0.5805	0.9712	0.7386	0.7981
IL12RB1	0.5671	-0.4951	0.8842	0.7213	0.7894
IL18BP	0.6788	-0.6081	0.9513	0.7964	0.7561
IL1B	0.6000	-0.5218	0.9016	0.6910	0.8276
IL2RG	0.6762	-0.5670	0.9558	0.7450	0.8570
IL7R	0.7830	-0.7219	0.9251	0.7111	0.7231
INPP4B	0.5282	-0.3684	0.7323	0.8413	0.3353
ITK	0.7129	-0.6407	0.9513	0.8534	0.6835
ITM2A	0.6112	-0.5166	0.6322	0.2964	0.8495
JAML	0.6204	-0.4842	0.8989	0.5941	0.9242
LAX1	0.5880	-0.4556	0.8995	0.8705	0.6124
LCK	0.6607	-0.5772	0.8526	0.4556	0.9332
LEF1	0.8368	-0.7406	0.9064	0.6567	0.7733

LSP1	0.7315	-0.6416	0.9512	0.7454	0.8357
LTB	0.5589	-0.4510	0.8120	0.3616	0.9579
MAP4K1	0.6026	-0.4965	0.9470	0.6951	0.8722
MCOLN2	0.7553	-0.6944	0.9611	0.7827	0.7768
MIAT	0.8501	-0.8095	0.8758	0.7257	0.6596
MICAL1	0.7798	-0.6629	0.9382	0.7063	0.8562
MIR155HG	0.5701	-0.5069	0.6526	0.2871	0.8665
MYO1G	0.6466	-0.5783	0.8956	0.7854	0.6962
NELL2	0.7427	-0.7069	0.9164	0.6305	0.8050
NFE2L3	0.6884	-0.6650	0.9230	0.7576	0.6918
NIBAN1	0.6504	-0.5587	0.8701	0.9190	0.5305
NLRC5	0.5838	-0.5354	0.8107	0.8671	0.5921
OR2I1P	0.6536	-0.5503	0.9472	0.7341	0.7933
ORAI2	0.6591	-0.5028	0.8246	0.6380	0.7417
P2RY10	0.6209	-0.5427	0.8080	0.6389	0.7844
P2RY14	0.7663	-0.6242	0.8956	0.6638	0.7591
PAPLN	0.5826	-0.5786	0.7770	0.5197	0.8392
PFKP	0.5552	-0.4521	0.8603	0.7617	0.7357
PKM	0.5414	-0.4943	0.8633	0.8392	0.6898
PLA2G2D	0.5800	-0.4682	0.9389	0.7327	0.7515

PLAU	0.5544	-0.4461	0.8458	0.6738	0.7391
PLCL1	0.5030	-0.4344	0.8002	0.7814	0.5112
PPP1R16B	0.5614	-0.5223	0.7021	0.8764	0.2505
PTPN22	0.5755	-0.5027	0.8489	0.8836	0.5471
PTPN7	0.6095	-0.5109	0.8428	0.4905	0.9667
PTPRE	0.6220	-0.5722	0.8684	0.9470	0.5690
PYHIN1	0.6852	-0.6882	0.8591	0.7085	0.7778
RARRES1	0.6523	-0.4984	0.9513	0.7542	0.7912
RASAL3	0.6770	-0.5797	0.8169	0.4182	0.9605
RASGRP1	0.6226	-0.5554	0.9234	0.8820	0.6004
RTN1	0.5501	-0.4416	0.8491	0.7417	0.6486
SAMHD1	0.4451	-0.4190	0.7179	0.9074	0.3977
SGPP2	0.6224	-0.6407	0.8376	0.8459	0.4968
SIGLEC8	0.7818	-0.6288	0.8449	0.5297	0.7324
SLAMF1	0.7003	-0.6165	0.9235	0.6195	0.8433
SLAMF7	0.6745	-0.5645	0.9636	0.8304	0.7514
SLAMF8	0.6956	-0.5625	0.9709	0.7588	0.8044
SLC1A5	0.7029	-0.5520	0.9159	0.7419	0.7741
SLC25A36	0.3745	-0.3712	0.6423	0.8755	0.2426
SPN	0.6255	-0.6332	0.8586	0.9301	0.4989
SPOCK2	0.6148	-0.5414	0.9178	0.8710	0.5922

STAT5A	0.5556	-0.5168	0.8405	0.6599	0.6780
SYTL1	0.5357	-0.4868	0.5234	0.0585	0.8311
TAGAP	0.5658	-0.4855	0.8974	0.8271	0.6350
THEMIS	0.6884	-0.5442	0.9147	0.8615	0.6660
TMEM65	0.5241	-0.5522	0.5051	0.8203	0.0495
TNF	0.6494	-0.5133	0.9366	0.6428	0.8535
TNFRSF25	0.6571	-0.4771	0.6974	0.3324	0.8329
TNFRSF9	0.7874	-0.6520	0.9161	0.7731	0.7714
TRAC	0.7393	-0.6727	0.9553	0.7055	0.8784
TRAF1	0.7494	-0.6725	0.9425	0.7872	0.7864
TRAF3IP3	0.6353	-0.4658	0.8710	0.6589	0.7616
TRBC1	0.5485	-0.4359	0.8205	0.4329	0.9561
TRBC2	0.5252	-0.4156	0.6588	0.1877	0.9410
UBASH3A	0.6718	-0.5531	0.9370	0.6965	0.8321
UBD	0.6021	-0.4974	0.9288	0.6095	0.8831
WDFY4	0.6334	-0.5614	0.8523	0.9027	0.5408
XCR1	0.7665	-0.6720	0.8338	0.7863	0.5427
ZAP70	0.7404	-0.6726	0.8323	0.5194	0.9231
ZC3H4	0.3079	-0.3052	0.5109	0.8384	0.0317
ZEB2	0.4104	-0.3812	0.5936	0.8972	0.1528

ZNF831	0.6464	-0.5604	0.9135	0.8228	0.6351

Table S4. KEGG functional enrichment analysis of core genes in bioinformatics.

ID	Description	GeneRatio	Gene	Count	Qvalue	*P*value	*P*.adjust
hsa04060	Cytokine-cytokine receptor interaction	20/78	TNFRSF9/IL12RB1/IL1B/CCR7/CXCL9/IL2RG/CCR5/IL7R/CXCL10/CXCL11 /XCR1/CCR4/CXCR3/TNF/CCL18/CXCR4/TNFRSF25/LTB/CCL4/CSF2RB	20	1.12E-10	8.79E-13	1.40E-10
hsa04061	Viral protein interaction with cytokine and cytokine receptor	13/78	CCR7/CXCL9/IL2RG/CCR5/CXCL10/CXCL11/XCR1/CCR4/CXCR3/TNF/ CCL18/CXCR4/CCL4	13	1.47E-10	3.46E-12	1.84E-10
hsa04640	Hematopoietic cell lineage	13/78	CD5/CD2/FLT3/IL1B/CD1E/CD3G/IL7R/GP1BA/CD3E/TNF/CD37/CD3D /CD8B	13	1.47E-10	3.04E-12	1.84E-10
hsa04660	T cell receptor signaling pathway	12/78	GRAP2/ITK/CD3G/RASGRP1/CD28/CD3E/TNF/ZAP70/CD3D/CD8B/LCK /CARD11	12	2.02E-08	6.36E-10	2.53E-08
hsa04062	Chemokine signaling pathway	12/78	ITK/CCR7/CXCL9/CCR5/CXCL10/CXCL11/XCR1/CCR4/CXCR3/CCL18 /CXCR4/CCL4	12	2.20E-06	1.21E-07	2.74E-06
hsa04064	**NF-kappa B signaling pathway**	10/78	TRAF1/**PLAU**/IL1B/TNF/ZAP70/BCL2A1/LCK/LTB/CCL4/CARD11	10	5.45E-07	2.57E-08	6.80E-07
hsa04659	Th17 cell differentiation	9/78	IL12RB1/IL1B/STAT5A/IL2RG/CD3G/CD3E/ZAP70/CD3D/LCK	9	7.39E-06	4.64E-07	9.22E-06
hsa05340	Primary immunodeficiency	8/78	IL2RG/IL7R/CD3E/ZAP70/CD3D/CD8B/LCK/CIITA	8	3.01E-08	1.18E-09	3.75E-08

hsa04658	Th1 and Th2 cell differentiation	8/78	IL12RB1/STAT5A/IL2RG/CD3G/CD3E/ZAP70/CD3D/LCK	8	2.15E-05	1.52E-06	2.69E-05
hsa05235	PD-L1 expression and PD-1 checkpoint pathway in cancer	7/78	CD3G/RASGRP1/CD28/CD3E/ZAP70/CD3D/LCK	7	0.0002	1.39E-05	0.0002

Table S5. KEGG functional enrichment analysis of altered genes caused by downregulating PLAU in macrophages in transcriptomics.

ID	Description	GeneRatio	Gene	Count	Qvalue	*P*value	*P*.adjust
rno04060	Cytokine-cytokine receptor interaction	7/44	Ccl3/Ccl6/Ccl4/Tnf/Tnfrsf25/Ccl22/Lifr	7	0.0037	9.54E-05	0.0046
rno04640	Hematopoietic cell lineage	5/44	Cd36/Itgb3/Tnf/Anpep/Siglech	5	0.0018	3.07E-05	0.0022
rno04061	Viral protein interaction with cytokine and cytokine receptor	5/44	Ccl3/Ccl6/Ccl4/Tnf/Ccl22	5	0.0018	1.97E-05	0.0022
rno04148	Efferocytosis	5/44	Cd36/Odc1/Abca1/Itgb3/Ptgs2	5	0.0109	0.0004	0.0133
rno04062	Chemokine signaling pathway	5/44	Ccl3/Ccl6/Ccl4/Ccl22/Rasgrp2	5	0.0111	0.0008	0.0135
rno03320	PPAR signaling pathway	4/44	Cd36/Fabp5/Acsbg1/Fabp4	4	0.0109	0.0005	0.0133
rno04620	Toll-like receptor signaling pathway	4/44	Ccl3/Ccl4/Tnf/Spp1	4	0.0111	0.0007	0.0135
rno04064	**NF-kappa B signaling pathway**	4/44	Ccl4/**Plau**/**Ptgs2**/Tnf	4	0.0111	0.0007	0.0135

rno04923	Regulation of lipolysis in adipocytes	3/44	Ptgs2/Ptgs1/Fabp4	3	0.0207	0.0016	0.0252
rno05140	Leishmaniasis	3/44	Nos2/Tnf/Ptgs2	3	0.0340	0.0032	0.0415

KEGG, Kyoto Encyclopedia of Genes and Genomes Enrichment Analysis. PLAU, urokinase-type plasminogen activator. Ptgs2, prostaglandin-endoperoxide synthase 2.

Table S6. Altered genes enriched in NF-κB signaling pathway after downregulating PLAU in macrophages in transcriptomics.

GeneUID	Gene	GeneAnno	Location (GRCm38.p6)	Blank1	Blank2	Blank3	KD1	KD2	KD3	Style	log2FC	FDR	-log10FDR	*P*value
ENSMUSG00000018930	Ccl4	chemokine (C-C motif) ligand 4 [Source:MGI Symbol;Acc:MGI:98261]	11:83662584-83664683	334	633	338	126	145	116	down	-1.4264	9.58E-09	8.0186	9.21E-12
ENSMUSG00000021822	**Plau**	plasminogen activator, urokinase [Source:MGI Symbol;Acc:MGI:97611]	14:20836660-20843385	2800	1904	1277	595	711	492	**down**	-1.4567	1.03E-05	4.9858	**3.36E-08**
ENSMUSG00000032487	**Ptgs2**	prostaglandin-endoperoxide synthase 2 [Source:MGI Symbol;Acc:MGI:97798]	1:150100031-150108227	644	861	562	244	364	232	**down**	-1.0083	1.23E-08	7.9103	**1.27E-11**
ENSMUSG00000024401	Tnf	tumor necrosis factor [Source:MGI Symbol;Acc:MGI:104798]	17:35199381-35202007	2733	6043	3401	1478	1650	1271	down	-1.1411	1.72E-05	4.7638	6.24E-08

NF-κB, Nuclear factor kappa B. PLAU,urokinase-type plasminogen activator. Ptgs2, prostaglandin-endoperoxide synthase 2.
